# PD-1 blockade attenuates immunosuppressive myeloid cells due to inhibition of CD47/SIRPα axis in HPV negative head and neck squamous cell carcinoma

**DOI:** 10.18632/oncotarget.5955

**Published:** 2015-11-07

**Authors:** Guang-Tao Yu, Lin-Lin Bu, Cong-Fa Huang, Wen-Feng Zhang, Wan-Jun Chen, J. Silvio Gutkind, Ashok B. Kulkarni, Zhi-Jun Sun

**Affiliations:** ^1^ The State Key Laboratory Breeding Base of Basic Science of Stomatology & Key Laboratory of Oral Biomedicine, Ministry of Education, Wuhan University, Wuhan, China; ^2^ Department of Oral Maxillofacial-Head Neck Oncology, School and Hospital of Stomatology, Wuhan University, Wuhan, China; ^3^ Oral and Pharyngeal Cancer Branch, Laboratory of Cell and Developmental Biology, National Institute of Dental and Craniofacial Research, National Institutes of Health, Bethesda, MD, USA; ^4^ Functional Genomics Section, Laboratory of Cell and Developmental Biology, National Institute of Dental and Craniofacial Research, National Institutes of Health, Bethesda, MD, USA

**Keywords:** HNSCC, myeloid-derived suppressor cell, tumor associated macrophagy, PD-1

## Abstract

Myeloid-derived suppressor cells (MDSCs) and tumor associated macrophages (TAMs) play key roles in the tumor immune suppressive network and tumor progression. However, precise roles of programmed death-1 (PD-1) in immunological functions of MDSCs and TAMs in head and neck squamous cell carcinoma (HNSCC) have not been clearly elucidated. In the present study, we show that PD-1 and PD-L1 levels were significantly higher in human HNSCC specimen than in normal oral mucosa. MDSCs and TAMs were characterized in mice and human HNSCC specimen, correlated well with PD-1 and PD-L1 expression. αPD-1 treatment was well tolerated and significantly reduced tumor growth in the HNSCC mouse model along with significant reduction in MDSCs and TAMs in immune organs and tumors. Molecular analysis suggests a reduction in the CD47/SIRPα pathway by PD-1 blockade, which regulates MDSCs, TAMs, dendritic cell as well as effector T cells. Hence, these data identify that PD-1/PD-L1 axis is significantly increased in human and mouse HNSCC. Adoptive αPD-1 immunotherapy may provide a novel therapeutic approach to modulate the micro- and macro- environment in HNSCC.

## INTRODUCTION

Head and neck squamous cell carcinoma (HNSCC), with more than 450,000 newly diagnosed cases every year, is the 6^th^ most common cancer worldwide [1]. As the oral cavity is the most frequent site (~90%) affected in HNSCC, it impacts highly critical functions of respiration, swallowing food and water as well as speech [2]. In developing countries, the tobacco-related human papillomavirus (HPV)-negative HNSCC has more morbidity and mortality than HPV-positive HNSCC [3]. HPV-negative HNSCC is usually accompanied by mutations of *p53* and loss of expression of *PTEN* [3, 4]. Although in recent years significant advances have been made in targeted therapies, HNSCC recurrence, resistance to chemo-radiotherapy and cervical lymph node metastasis persist as the most important factors affecting the poor prognosis of patients, particularly in refractory HPV-negative HNSCC. Therefore identification and characterization of the molecular mechanisms underlying HNSCC initiation and progression are *a priori* for timely diagnosis and developing effective treatment.

Various mechanisms have been proposed for the resistance of HNSCC to immune recognition and response, including recruitment of myeloid derived suppressor cells (MDSCs), tumor associated macrophages (TAMs), regulatory T cells (Tregs), and local secretion of “alternatively activated” immunosuppressive soluble factors such as TGFβ1, IL10 and IL13 [5]. Recent advances in therapeutic antibodies, cancer vaccines, and adoptive T-cell therapy (ACT) have shown promising therapeutic potential of immunotherapy in treating patients with cancer [6]. Tumor-mediated immunosuppression is also considered to be a major barrier for successful cancer immunotherapy. Recent evidence has suggested that tumor-mediated immunosuppression by the up-regulation of coinhibitory immune checkpoints such as programmed death 1 (PD-1) and cytotoxic T-lymphocyte antigen 4 (CTLA-4) represent major obstacles to the generation and maintenance of clinically meaningful antitumor immunity [7, 8].

PD-L1 (a principal ligand of PD-1), known to be expressed by cells in the tumor microenvironment, engages PD-1 on T cells and subsequently triggers inhibitory signaling, downstream of the T-cell receptor, blocking effector functions and reducing the T-cell killing capacity [9]. PD-L1 can be constitutively expressed on the surface of cancer cells through poorly characterized oncogenic signaling pathways [10, 11]. PD-L1 is also expressed in immune cells in response to the presence of immune-stimulating cytokines [12]. The important role of PD-1/PD-L1 axis in the tumor immunosuppressive effect stems from recent clinical trials of PD-1 blockade that resulted in significant survival benefit with minimal toxicity to patients with advanced melanoma, renal cell carcinoma, and non–small cell lung cancer [13–16].

In the current study, we report that significant increase in PD-1/PD-L1 expression is an important immunosuppressive mechanism in human and mouse HNSCC. Oncogene activation by the conditional knockout of *Pten* and *Tgfbr1* may contribute to the over-expression of PD-L1 with concomitantly significant increase in MDSCs and TAMs. Moreover, we discovered that the blockade of PD-1 significantly reduces CD11b^+^Gr1^+^ and CD11b^+^ F4/80^+^ cells in immune organs as well as in tumors of the mouse model. Our study, in direct relevance to clinical application, demonstrates that targeting PD-1/PD-L1 can lead to durable antitumor immunity and curative outcome, with remarkable reduction in MDSCs and TAMs followed by enhanced immunoreactivity of CD8^+^ T and CD4^+^ T cells. These findings will be valuable in developing good strategies aimed at achieving more effective immunotherapy to treat HNSCC.

## RESULTS

### Increased expression of PD-1/PD-L1 in human HNSCC

**Figure 1:**
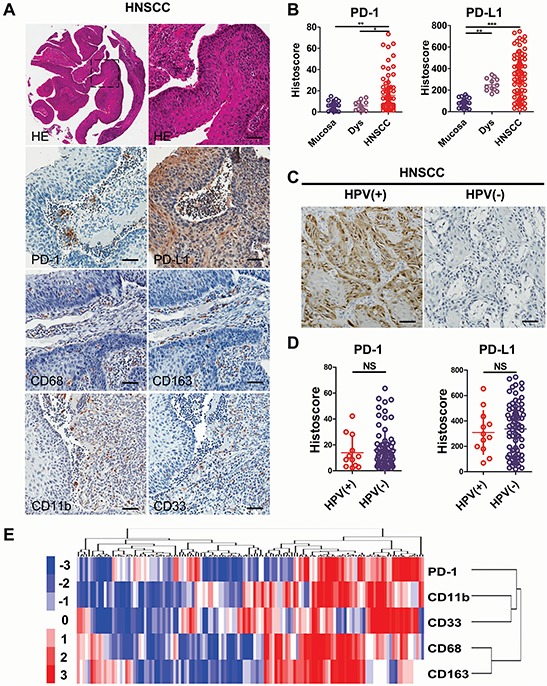
High expression of PD-1/PD-L1 in human head neck squamous cell carcinoma **A.** representative Hematoxylin-eosin (HE) and immunohistochemistry staining of PD-1, PD-L1, CD68, CD163, CD11b and CD33 in HNSCC tissue. Scale bar, 50 μm. **B.** quantification of immunohistochemical histoscore of PD-1 and PD-L1 among oral mucosa (*n* = 32), dysplasia (Dys, *n* = 12) and head neck squamous cell carcinoma (HNSCC, *n* = 86). (All data are presented as mean ± SEM, One way ANOVA with post Tukey test. **P < 0.01; ***P < 0.001). **C.** representative immunohistochemistry staining of p16 to identify HPV + (*n* = 12) and HPV- (*n* = 74) HNSCC. Scale bar, 50 μm. **D.** PD-1 and PD-L1 histoscore of HPV+ and HPV- in HNSCC. NS, No significance. **E.** hierarchical clustering of PD-1, CD68, CD163, CD11b and CD33 immunohistochemical results in human HNSCC, statistic including mucosa, dysplasia and HNSCC (*n* = 130).

To determine whether PD-1/PD-L1 expression was associated with HNSCC in humans, we searched the publicly available dataset of cancer using the Oncomine database [17]. In a meta-analysis of 18 datasets of head and neck cancers gene expression profiling, the increased *CD274* (gene encoding PD-L1) and CD279 (gene encoding PD-1) DNA copy number, as well as increased mRNA expression of this genes, was significantly increased in HNSCC as compared with the controls (*P* < 0.05, Fig. S1A–S1C). To evaluate PD-1/PD-L1 levels in human HNSCC tissues, we performed immunohistochemistry in human HNSCC sections (Fig. 1A). PD-1 immunostaining revealed elevated levels in inflammatory cells of the cancerous tissue, and in particular in the invasive front of the tumor (Fig. 1A). PD-L1 immunostaining displayed its predominant expression in membrane and cytoplasm of HNSCC cells. There was significantly increased immunostaining for PD-1/PD-L1 in human HNSCC (*n* = 86) as compared with dysplasia (*n* = 12) and normal oral mucosa (*n* = 32) (Fig. 1B).

### Increased PD-1/PD-L1 expression in HNSCC tissue is independent of HPV status

A recent interesting report identified the possible over-expression of PD-1/PD-L1 in human HNSCC, particularly in human papilloma virus (HPV) positive patients. However, in the present patient cohort the main anatomy origin of HNSCC was from oral cavity. Using p16 immunostaining and HPV DNA *in situ* hybridization technique to monitor HPV infection, we found that there was no statistically significant difference in PD-1/PD-L1 expression in HPV+ (*n* = 12) and HPV- (*n* = 74) HNSCC (Fig. 1C and 1D). This result prompted us to explore whether there was a difference in PD-1 and PD-L1 expression in other viral-associated cancer settings. We searched for viral related cancer datasets including TCGA data using the Oncomine database. Our findings suggested that there are no significant differences in the CD279/CD274 DNA copy number or the mRNA levels in HPV-related HNSCC or HPV-related cervical cancer (Fig. S2 and S3). Additionally, there is no difference in either CD279 or CD274 expression in Epstein-Barr virus-related lymphoma or Hepatitis B virus/Hepatitis C virus-related hepatocellular carcinoma (Fig. S2 and S3). These results indicated that PD-1 over-expression potentially play an important role in both HPV+ and HPV- HNSCC.

### PD-1/PD-L1 protein levels correlate with MDSCs and TAMs in human HNSCC

Recent studies have demonstrated that MDSCs and TAMs are predominantly cancer immune suppressor cells [5], which prompted our study on the correlation of PD-1/PD-L1 with MDSCs and TAMs. Immunostaining with specific antibodies for CD68 and CD163, were used to stain the M2 type macrophages in human HNSCC tissue array. As shown in Fig. 1A, an increase in CD68 and CD163 expression in the tumor areas of HNSCCs compared with normal mucosa were observed (see quantification in Fig. S4A). Additionally, specific antibodies for CD11b and CD33 were used to stain the immune cells which are similar to mice Gr1^+^CD11b^+^ MDSCs (see quantification in Fig. S4A). The expression of CD68, CD163, CD11b and CD33 was not related with the HPV+ status (Fig. S4B). By hierarchy clustering and linear regression, we found that the expression of PD-1 was closely related to the expression of CD11b, CD33, CD68 and CD163, and in particular closely to CD11b and CD33 (Fig. S4C and Fig. 1E). These observations suggest that PD-1/PD-L1 are closely related to the TAMs as well as MDSCs in human HNSCC. PD-1/PD-L1 expression and the TAMs or MDSCs population were increased in both HPV+ and HPV- HNSCC.

### The loss of tumor suppressor genes increases the expression of PD-L1 with TAMs and MDSCs in mice

**Figure 2:**
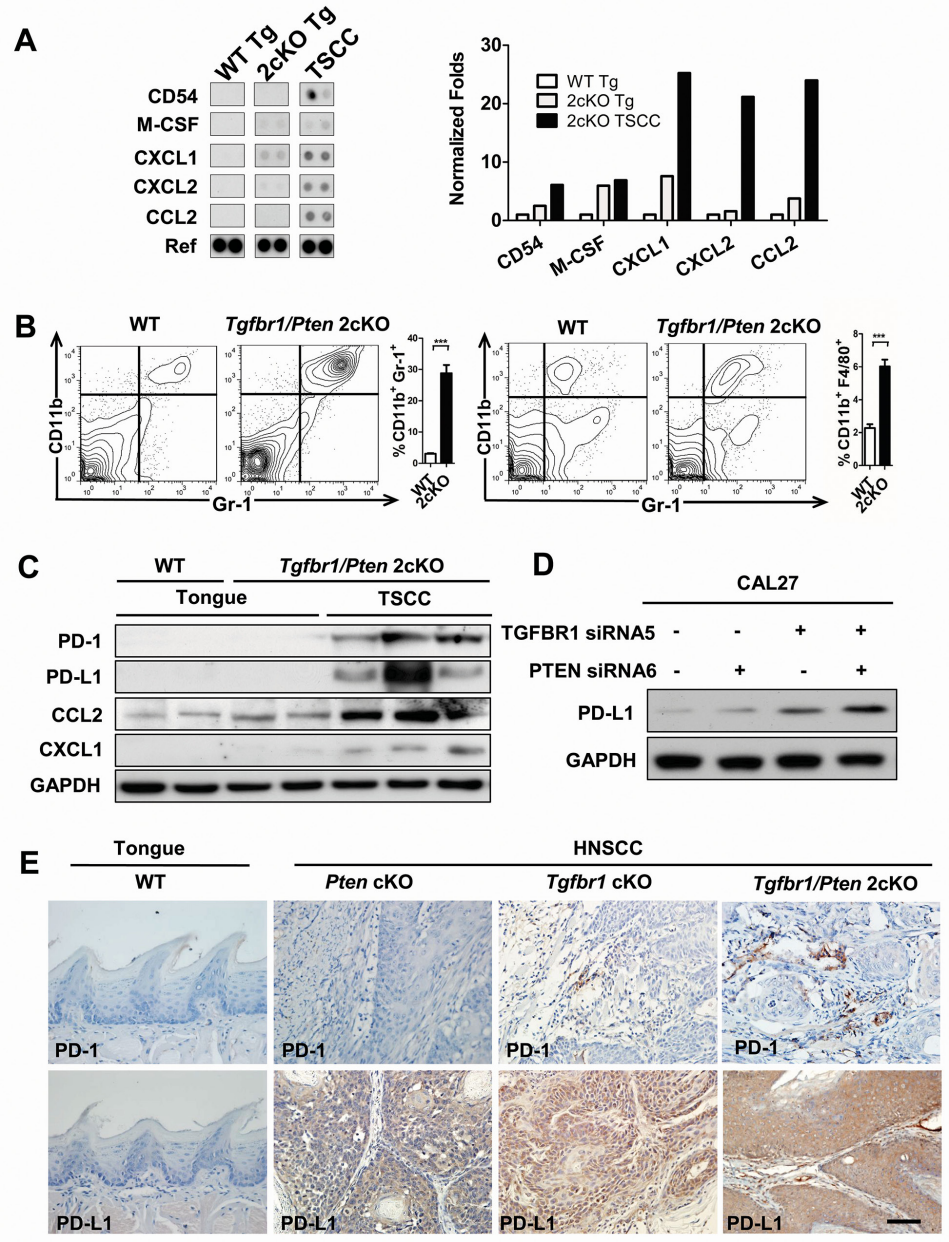
Combined deletion of *Pten* and *Tgfbr1* increased expression of PD-1/PD-L1 induced immune suppression status in mice HNSCC **A.** antibody array (left) shows increase of CD54, M-CSF, CXCL1, CXCL2 and CCL2 in *Tgfbr1/Pten* 2cKO mice tongue squamous cell carcinoma (TSCC) as compared with *Tgfbr1/Pten* 2cKO tongue (Tg) and wide type tongue (WT Tg, *n* = 5 mice respectively). Quantitative of dot blotting by normalized with housekeep genes (Ref) using Image J (right). **B.** there was significant difference between the expression level of CD11b^+^/Gr1^+^ and CD11b^+^/F4/80^+^ cell population in the spleen of tumor bearing mice as compared with spleen of wide type mice. (*n* = 6 mice respectively, *t* test, ***P < 0.001). **C.** western blot indicate increase of PD-1, PD-L1, CCL2 and CXCL1 in *Tgfbr1/Pten* 2cKO mice tongue squamous cell carcinoma (TSCC) as compared with *Tgfbr1/Pten* 2cKO tongue (Tg) and wide type tongue (WT Tg). GAPDH was used as the internal protein loading control. Experiment were repeated twice. **D.** siRNA knock down assay suggest loss function of *Tgfbr1* and *Pten* increase expression of PD-L1 in CAL27 HNSCC cell line. Experiment were repeated twice and performed in another cell line. **E.** immunohistochemical staining indicate increase PD-1 and PD-L1 expression in *Tgfbr1* conditional knock out (*Tgfbr1* cKO, *n* = 5) mice HNSCC, *Pten* conditional knock out (*Pten* cKO, *n* = 5) mice HNSCC and *Tgfbr1/Pten* 2cKO (*n* = 5) mice HNSCC, Data presented as mean ± SEM, One way ANOVA with post Tukey test. Scale bars = 50um.

Our previous work reported a spontaneous development of HNSCC in a mouse model by combined deletion of important tumor suppressors *Pten* and *Tgfbr1* [18]. The tumor bearing mice showed significantly increased expression of CD11b+ and Gr1+ cell populations in tumors and immune organs. To detect multiple cytokines and chemokines in the mouse HNSCC, we performed mouse cytokine antibody array which profiles the expression of 40 key cytokine genes involved in inflammation of mouse HNSCC tissues (Fig. 2A). Surprisingly, we found that most significantly increased cytokines such as sICAM-1 (CD54), M-CSF CXCL1, CXCL2, CCL2 in the *Tgfbr1/Pten* 2cKO HNSCC may attract monocytes. By quantitative PCR we found that indeed the expression of important cytokines Cd54, Csf-1, Cxcl1, Cxcl2 and Ccl2 was significantly increased in 2cKO mouse HNSCC tumor lysates as compared with 2cKO mouse tongue and the control tongue (Fig. S5: *n* = 5 respectively). We also found a remarkable increase in CD11b^+^Gr1^+^ MDSCs (Fig. 2B: *n* = 6 respectively, *P* < 0.001) as well as CD11b^+^F4/80^+^ TAMs (Fig. 2B: *n* = 6 respectively, *P* < 0.001) in *Tgfbr1/Pten* 2cKO HNSCC. We further confirmed increased expression of PD-1, PD-L1 with CCL2 and CXCL1 in the tumor lysaytes of 2cKO mice as compared with syngeneic counterparts (Fig. 2C). A recent report indicated that activation of oncogene Akt increases the expression of PD-L1. Thus, we explored the exact role of loss of function of *Pten* or *Tgfbr1* in PD-L1 expression. Western blot results consistently suggested that the loss of *PTEN* and/or *TGFBR1* increased expression of PD-L1 in human head neck cell line CAL27 (Fig.2D) and FaDu (Fig. S6). This interesting finding has also been confirmed by the observation of increased PD-L1 expression in mouse HNSCC of *Pten* conditional knockout (*Pten* cKO) mice, *Tgfbr1* conditional knockout (*Tgfbr1* cKO) mice and *Pten/Tgfbr1* 2cKO mice (Fig. 2E).

### Blockade of PD-1 prevents tumorigenesis in a HNSCC mouse model

**Figure 3:**
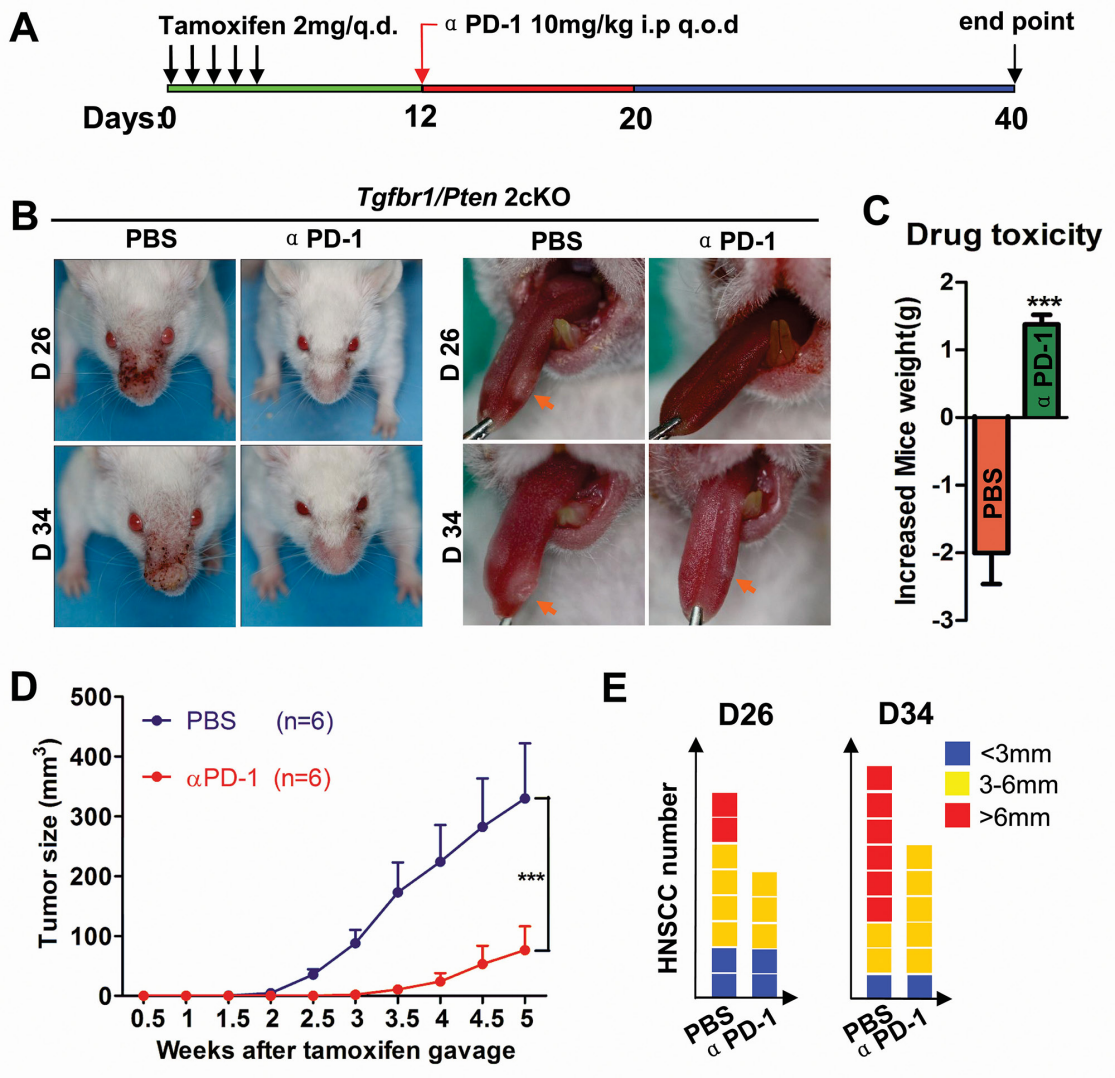
Inhibition of PD-1 by RMP1–14 prevents tumorigenesis and delays the onset of HNSCC in genetically-engineered HNSCC mouse models **A.** *Tgfbr1/Pten* 2cKO mice bearing carcinoma by tamoxifen were treated with αPD-1 intraperitoneally (i.p) every other day for 10 days or vehicle control treated (*n* = 6 mice respectively). **B.** representative photos of mice with external head and neck tumor (left panel) and tongue tumor (right panel) after treatment with αPD-1 or vehicle in day 26 and day 34 after tamoxifen gavage. **C.** drug toxicity was assessed by gained body weight of *Tgfbr1/Pten* 2cKO mice in each group. **D.** total tumor volume was assessed in αPD-1 and control group twice a week after tamoxifen gavage. Data presented as mean ± SEM (unpaired Student *t* test, ***P < 0.001). **E.** the number of tumor and the volume of each tumor were measured after treatment with αPD-1 or vehicle in day 26 and day 34 after tamoxifen gavage.

To determine the impact of PD-1 on carcinogenesis *in vivo*, we utilized *Tgfbr1/Pten* 2cKO mouse HNSCC model for chemopreventive tumorigenesis studies. The initial dosage of the inhibiting PD-1 antibody was applied at day 12. As the experimental paradigm shown in Fig. 3A, we found that PD-1 blockade produced a decrease in tumor growth, which indicated that *in vivo* inhibition of PD-1 using RMP1–14 antibody (10mg/kg intraperitoneally every other day) will delay (Fig. 3B) tumorigenesis in head and neck area as well as in tongue of the immunocompetent mice. The PD-1 blockade did not cause additional cytotoxicity as judged by gain of body weight in the treated mice as compared to the vehicle only group (*n* = 6 respectively, *P* < 0.001, Fig. 3C). Additionally, the head and neck tumor growth curve and the number and size of the tumor demonstrated that PD-1 blockade effectively reduced the tumor burden and number of head neck tumors (Fig. 3D and 3E).

### Blockade of PD-1 decreases MDSCs and TAMs in HNSCC mouse model

**Figure 4:**
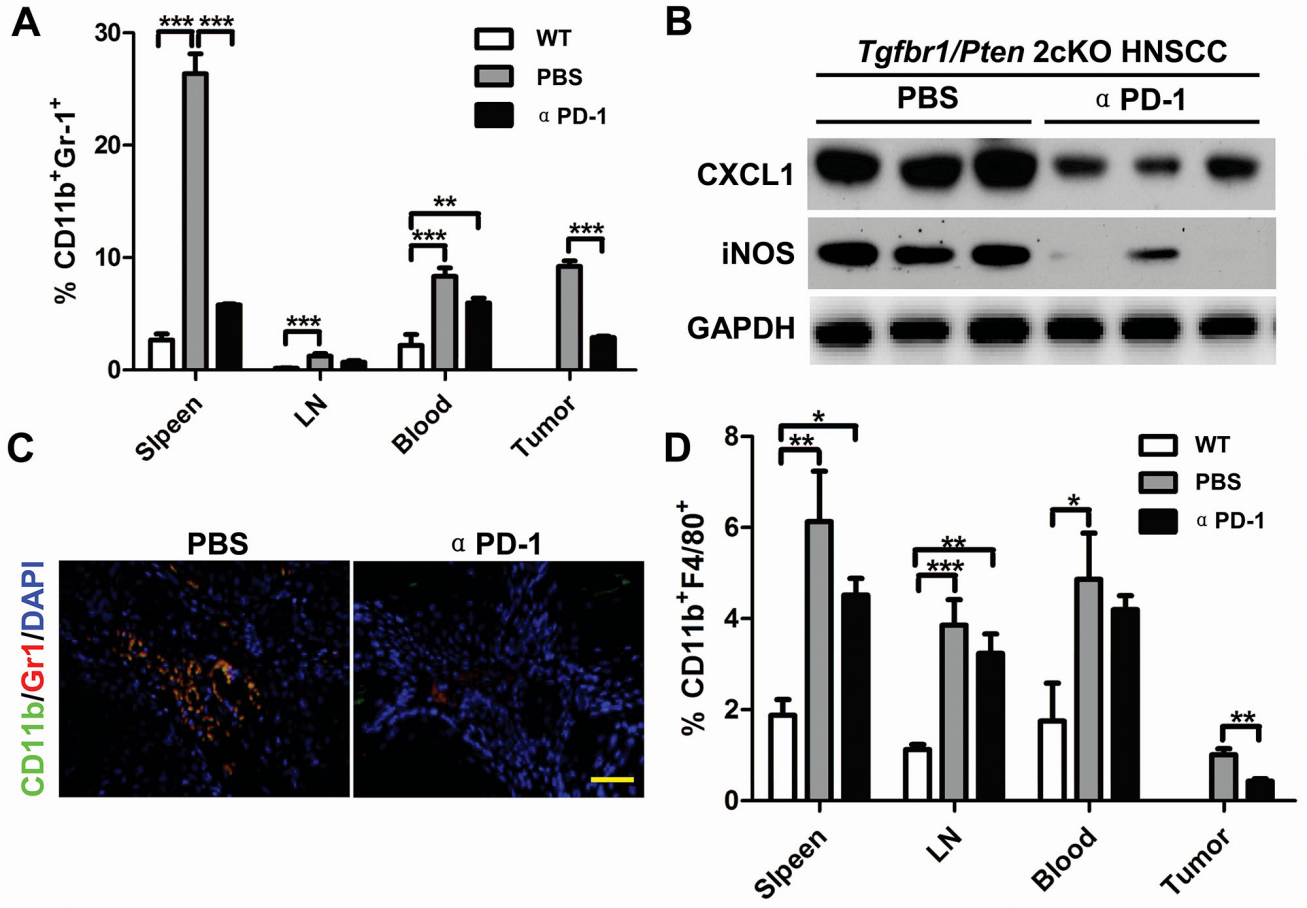
αPD-1 significantly attenuate increased CD11b+Gr1+ MDSCs and CD11b+F4/80+ TAMs in HNSCC bearing *Tgfbr1/Pten* 2cKO mouse model **A.** quantification the percent of CD11b^+^Gr1^+^ MDSCs in spleen, lymph nodes, blood and tumor of mice bear tumor with or without αPD-1 treatment and wide type mice (Data presented as mean ± SEM, *n* = 6 mice respectively, ANOVA with post Tukey test. **P < 0.01; ***P < 0.001). **B.** western blot analysis revealed that the protein level of CXCL1 and iNOS were reduced in different degrees with PD-1 inhibition. GAPDH was used as a loading control. Data are representative of two independent experiments **C.** double immunofluorescence staining of CD11b^+^ Gr1^+^ cell population in mice HNSCC with or without αPD-1 treatment. Scale bar, 50 um. **D.** quantification of CD11b^+^F4/80^+^ tumor associated macrophage (TAMs) in spleen, lymph nodes, blood and tumor of mice bear tumor with or without αPD-1 treatment and wide type mice (Data presented as mean ± SEM, *n* = 6 mice respectively, ANOVA with post Tukey test. *P > 0.05; **P < 0.01; ***P < 0.001).

To determine whether PD-1/PD-L1 blockade decreases the MDSCs as well as TAMs, we performed flow cytometry analysis on the cells from the spleen, lymph node, and blood as well as tumor tissue of *Tgfbr1/Pten* 2cKO mice with or without αPD-1 treatment. We found that PD-1 blockade remarkably reduced CD11b^+^Gr1^+^ MDSCs in spleen, lymph node, blood as well as in tumor as compared with the vehicle only and wild-type syngeneic mice (Fig. 4A and Fig. S7A). The CD11b^+^Gr1^+^ MDSCs are even significantly attenuated in spleen and tumor. Western blot analysis showed a significant decrease in CXCL1 and iNOS levels in tumor lysates of αPD-1 blockade group as compared with vehicle only group (Fig. 4B). Immunofluorescence assay further showed a significant decrease in Gr1^+^CD11b^+^ double positive MDSCs cell population as compared with control group (Fig. 4C and Fig. S7B). These results support our hypothesis that PD-1 blockade can effectively decrease MDSCs and TAMs *in vivo*.

In addition to MDSCs, we also investigated CD11b^+^F4/80^+^ TAMs in αPD-1-treated 2cKO mice as compared with vehicle only group. The flow cytometry analysis showed similar results to those seen in MDSCs. Tumor bearing mice had a significant increase in TAMs that were significantly attenuated by αPD-1 treatment particularly in spleen as well as in the tumors (Fig. 4D and Fig. S8).

### Inhibition of PD-1 reverses the immune suppression by promoting maturation of dendritic cells and increase in CD8^+^ and CD4^+^ T cell *in vivo*

**Figure 5:**
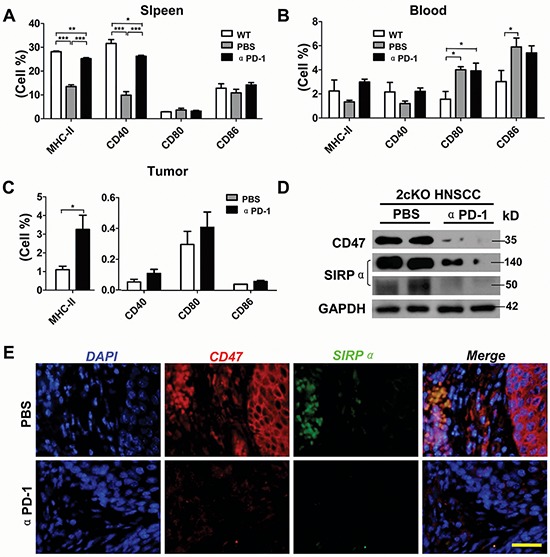
Inhibition of PD-1 significantly promotes maturation of DCs in HNSCC bearing *Tgfbr1/Pten* 2cKO mouse model Percentage of MHC-II, CD40, CD80 and CD86 positive cells was determined by flow cytometry in spleen **A.** and lymph node **B.** from mice tumor with or without αPD-1 treatment and wide type mice (Data presented as mean ± SEM, One way ANOVA with post Tukey test, *n* = 6 mice respectively, *P < 0.05; **P < 0.01; ***P < 0.001). **C.** percentage of MHC-II, CD40, CD80 and CD86 positive cells was determined by flow cytometry in twice bearing tumor with or without αPD-1 treatment (*t* test, *P < 0.05). **D.** western blot analysis of CD47 and SIRPα levels in mice bearing tumor with or without αPD-1. Data are representative of two independent experiments. **E.** representative immunofluorescence images of CD47 and SIRPα in mice bearing tumor with or without αPD-1 treatment are shown. Scale bar, 50 um.

The balance between effector immune cells such as CD8^+^ cytotoxic T cells and CD4^+^ “helper” T cells) and suppressor immune cells such as Tregs and MDSCs is a critical determinant of effective anti-tumor activity. We sought to determine the effect on dendritic cells (DCs) and T cells and found that PD-1 blockade significantly increased matured DCs population as well as increased CD8^+^ and CD4^+^ T cells. In tumor bearing *Tgfbr1/Pten* 2cKO mice, PD-1 blockade increased MHC-II^+^, CD40^+^, CD86^+^ cells in spleen (Fig. 5A and Fig. S9). In blood of tumor bearing *Tgfbr1/Pten* 2cKO mice, PD-1 blockade increased MHC-II^+^ and CD40^+^cells (Fig. 5B and Fig. S9). In tumor, inhibition of PD-1 effectively augmented CD40^+^, CD80^+^, CD86^+^ cells, and especially significantly increased MHC-II^+^ DCs (Fig. 5C and Fig. S9). A recent report indicates that CD47/SIRPα axis plays an important role by reducing mature DCs and attracting tumor associate macrophages [19]. Our data suggested that CD47 and SIRPα significantly increased in tumor but this was attenuated by PD-1 blockade (Fig. 5D). The immunofluorescence analysis also indicated that CD47 was over expressed in both cancer cells and inflammatory cells. SIRPα was mainly expressed in inflammatory cells and overlapped with CD47^+^ cell in peritumor inflammatory cells. PD-1 blockade decreased the expression of CD47 and SIRPα (Fig. 5E).

**Figure 6:**
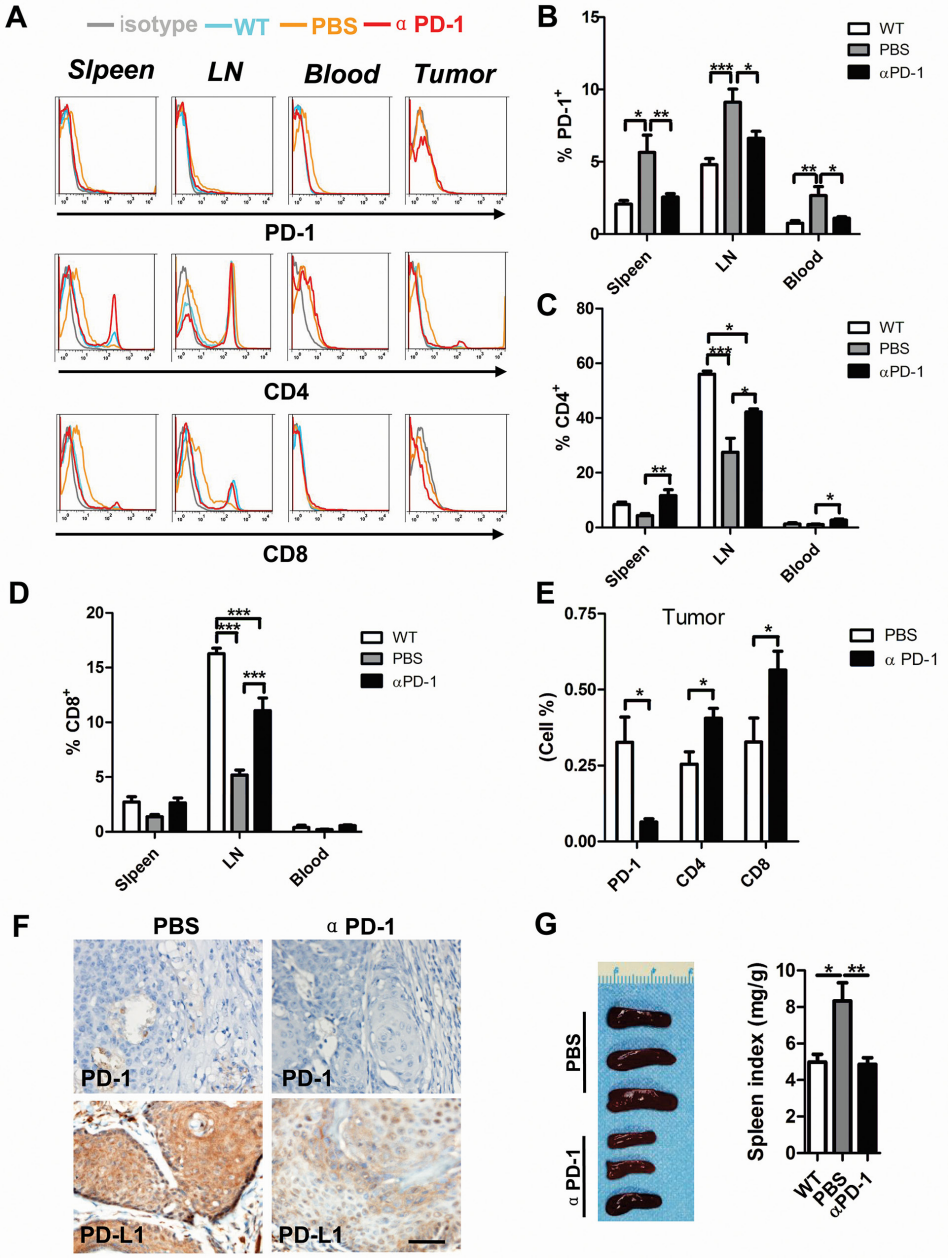
PD-1 blockade significantly increase CD4+ and CD8+ T cell in HNSCC mouse model **A.** representative flow cytometry showed decrease staining population of PD-1^+^ and increase of CD4^+^ and CD8^+^ T cell in αPD-1 treatment group. Quantitative of PD-1^+^ **B.** CD4^+^ **C.** CD8^+^ **D.** cell population in spleen, lymph node (LN) and blood of wild type mice, *Tgfbr1/Pten* 2cKO mice with or without αPD-1 treatment (*n* = 6 mice respectively, One way ANOVA with post Tukey test. *P < 0.05; **P < 0.01; ***P < 0.001). **E.** quantitative of PD-1^+^, CD4^+^ and CD8^+^ cell population in mice bearing tumor with or without αPD-1 treatment (*t* test, *P < 0.05). **F.** representative images demonstrates the immunohistochemical analysis of PD-1 and PD-L1 in mice bearing tumor with or without αPD-1 treatment. Scale bar, 50um. **G.** representative gross photo (left) showed the size of spleen different between αPD-1 treatment group and vehicle group. Spleen index shows αPD-1 significantly inhibit compensatory growth in *Tgfbr1/Pten* 2cKO mice. (Data presented as mean ± SEM, *n* = 6 mice respectively, right panel, One way ANOVA with post Tukey test. *P < 0.05; **P < 0.01).

To further investigate the number of CD4^+^ and CD8^+^ T cells, we took advantage of flow cytometry and found inhibition of PD-1 significantly decreased PD-1^+^ cells in spleen, lymph node as well as in blood (Fig. 6A and 6B), accompanied by an increase in CD4^+^ (Fig. 6A and 6C) and CD8^+^ (Fig. 6A and 6D) T cells in spleen, lymph node and blood. In tumors, PD-1 blockade significantly decreased PD-1^+^ cells accompanied by an increase in CD4^+^ and CD8^+^ T cells (Fig. 6A and 6E). Then, we investigated the expression of PD-1 and PD-L1 in mouse tumors with or without αPD-1 treatment (Fig. 6F). The reversal of immune tolerance status by PD-1 blockade can also be indicated by observation of reduced spleen size in αPD-1 group as compared with vehicle only group (Fig. 6G). Overall, these results suggest that the prior to the treatment peripheral effector /suppressor balance in PD-1 blocker treated mice is already skewed towards immunosuppression.

## DISCUSSION

Suppression of the host immune system plays a major role in cancer progression. The activation of the co-inhibitory checkpoint molecule PD-1 in T cells and expansion of MDSCs are considered the major mechanisms for tumor to escape from immune surveillance [20]. Although targeted PD-1/PD-L1 therapy achieved a significant cure in melanoma and lung cancer [21, 22], the importance of this axis in HNSCC is less recognized. For the first time, we demonstrate here that the increase of PD-1/PD-L1 axis correlates with expansion of key suppressor immune cell populations, MDSCs and TAMs, in human HNSCC as well as in HNSCC mouse model that develops spontaneous tumors. Moreover, our study provides direct evidence that systematic application of αPD-1 monoclonal antibody demonstrates a remarkable decrease in tumorigenesis with significantly reduced MDSCs and TAMs populations and increased effector T cells. Molecular analysis identified that αPD-1 treatment significantly decreases myeloid cells and chemokine levels as well as decrease in CD47/SIRPα signaling.

Alcohol/tobacco abuse and HPV infection are the main risk factors associated with the development of HNSCC [23, 24]. Although HPV- HNSCCs are different from HPV+ HNSCC in several ways [25, 26], there is no significant difference in PD-1/PD-L1 expression between HPV+ and HPV- HNSCC [27, 28]. Our data further demonstrate that MDSCs and TAMs frequency is independent of HPV infection status. As compared with HPV+ tumors, *PTEN* loss was found to be more frequent in HPV- HNSCC [25]. We found *PTEN* loss promotes the expression of PD-L1 in HNSCC cell line and in the conditional *Pten^−/−^* mice, which is consistent with recent observations in breast and prostate cancers [29]. The present study also suggests that *TGFBR1* loss further enhances the expression of PD-L1 in HNSCC cell lines and conditional *Tgfbr1*^−/−^ mice, which indicate that loss of *TGFBR1* may be an crucial molecular event leading to the accumulation of PD-L1 in both HPV infection and alcohol or tobacco use [18, 30]. Moreover, *PTEN* loss and *TGFBR1* loss synergistically promote activation of PD-L1, likely by the activation of oncogenic Akt pathway in *Tgfbr1/Pten* 2cKO mice [31]. Taken together, our *in vitro* and *in vivo* studies revealed that loss of *PTEN* and *TGFBR1* together promote the activation of PD-1 and PD-L1 pathway in HNSCC, independent of the HPV infection status.

More recent reports have suggested the close relationship of PD-1/PD-L1 with MDSCs and TAMs [32]. Our previous [18] and present study together reveals a significant accumulation of MDSCs and TAMs along with increase in putative myeloid cytokines and chemokines in HNSCC tumorigenesis in mice. We further confirmed that increased expression of PD-1, PD-L1 in HNSCC correlated with accumulation of MDSCs and TAMs in human and mouse HNSCC. As expected, PD-1 blockade significantly reduced tumorigenesis in *Tgfbr1/Pten* 2cKO mice. Moreover, a remarkable reduction of MDSCs cell populations in tumor and immune organs by PD-1 blockade was observed, which can be attributed to the decrease of CXCL1. The expression of iNOS in MDSC has been shown to be critical for the suppression of T cell [33]. Therefore, the reduction in iNOS further promotes the activation of T cell in tumor microenvironment. PD-1 has also been implicated in the regulation of M2 macrophages and inhibition of PD-1 can induce the polarization of macrophages to M1 phenotype [34]. The current study first outlines that PD-1 blockade has an antitumor function in HNSCC, at least partially, dependent on the role of CD47/SIRPα which functions as a “do not eat me” signal in many malignancies. Blockade of CD47/SIRPα axis led to efficient and rapid phagocytosis of multiple tumor cell types [35]. In our *in vivo* study, down-regulation of expression of CD47 in tumor cells by blockade PD-1 may improve phagocytosis of macrophage to enhance antitumor immunity.

Analogous to macrophages, the current study also demonstrated αPD-1 treatment promotes maturation of DCs. With exposure to antigens stimuli, DCs undergo a maturation process by increasing expression of co-stimulatory molecules such as MHC-II, CD40, CD80 and CD86, producing pro-inflammatory cytokines and presenting antigens to T cells [36]. Indeed, blockade of PD-1 increased the expression levels of co-stimulatory molecular in mouse tumor and immune organ suggesting the condition of immune suppression was improved in the tumor microenvironment, which may be caused by decreased CD47/SIRPα signaling. Consistently, recent reports indicated that activation of the CD47/SIRPα signaling pathway enables to restrain DCs maturation [19] and that inhibition of CD47/SIRPα may increase DCs-mediated antigen presentation [37]. The results in our study, especially in spleen and blood, demonstrated that the mechanisms by which tumor escape immune survelience depend on the tumor itself but also in changing conditions in tumor macro-environment [38].

PD-1/PD-L1 plays an important role in inhibiting tumor immune response by promoting CD4^+^ and CD8^+^ T cells apoptosis [39]. In addition to directly reducing CD4^+^ and CD8^+^ T cells apoptosis, PD-1 blockade may also restore T cell activity by decreasing the CD47 expression in the tumor microenvironment [40]. At the same time, DCs that could be partially activated by co-stimulatory molecules in the tumor microenvironment could then promote effective antitumor T cells responses [41]. The current study also revealed that blockade of PD-1 could decrease arginase 1 and iNOS which enhance T cells proliferation to promote antitumor immunity [42, 43]. We have also demonstrated a significant correlation between PD-1 expression and infiltration of CD11b^+^/CD33^+^ (markers of human MDSCs) cells in human HNSCC tissues microarray.

The clinical significance of PD-1 blockade for HNSCC treatment is supported by phase III single agent clinical trial of PD-1 antibody Nivolumab (BMS-936558, NCT02105636; see review of Zandberg DP) [44]. Anti-PD-1 mAB appears to be generally less toxic as compared to anti-CTLA4 mAB, with a slightly different toxicity profile such as pneumonitis [45]. To date, there are not too many general adverse events reported for anti-PD-1, including fatigue, pyrexia, chills and infusion reaction [46]. The current study suggests that targeting the PD-1/PD-L1 signaling pathway to reverse the immunosuppressive status has important therapeutic implications for the treatment of HNSCC. Nevertheless, we cannot completely exclude the possibility that PD-1 blockade may have indirect role on tumorigenesis through angiogenesis and by targeting cancer stem cell mediated by MDSCs and TAMs [47]. In addition, a recent report indicated increased MDSCs [16] and PD-1^+^ CD4 T cells [48] in cancer patients receiving chemo-radiotherapy, which provides a rationale for combined treatment with chemo-radiotherapy and PD-1 blockade.

In summary, we discovered a significant increase of PD-1/PD-L1 in HNSCC, which is tightly correlated with important immune suppressor cells, MDSCs and TAMs. Taking advantage of immunocompetent transgenic mouse HNSCC models, we observed a decrease in MDSCs, TAMs, and mature DCs as well as an increase in CD8^+^ T cells and CD4^+^ T cells by PD-1 blockade in micro- and macro- tumor environment. Therefore, we believe that therapeutically blocking the PD-1/PD-L1 pathway may prove to be a promising antitumor therapy for HNSCC.

## MATERIALS AND METHODS

Detailed methods and procedures are provided in the Supplementary Data.

### Genetically modified mice

All experiments were conducted in accordance with the guidelines of the Institutional Animal Care and Use Committee of the Wuhan University. Time inducible tissue-specific *Tgfbr1/Pten* 2cKO mouse (*K14-Cre*^ERtam+/−^; *Tgfbr1*^flox/flox^; *Pten*^flox/flox^) were maintained and genotyped according to published protocols [49, 50]. The details of *Tgfbr1* cKO HNSCC mouse (*K14-Cre*^ERtam+/−^; *Tgfbr1*^flox/flox^), *Pten* cKO HNSCC mice (K14-Cre^ERtam+/−^; *Pten* ^flox/flox^) were previously described [49, 50]. All the mice were bred in the FVBN/CD1/129/C57 mixed background.

### PD-1 antibody treatment

The *in vivo* MAb *anti* mPD1 antibody (RMP1–14) was purchased from BioXcell (West Lebanon, NH, USA) After oral gavage of tamoxifen for 5 consequent days, the *Tgfbr1/Pten* 2cKO mice were randomly divides into control group (PBS, i.p. daily, *n* = 6 mice), 10 mg/kg RMP1–14 treated group (i.p. daily: *n* = 6 mice). RMP1–14 and vehicle treatment were performed 10 days after tamoxifen induction and the mice were observed for 18 days. Syngeneic wide type control mice (*K14-Cre*^ERtam+/−^; *Tgfbr1*^flox/flox^; *Pten*^flox/flox^) treated with the same dose of tamoxifen were used as controls (*n* = 6). The tumor sizes were measured using a micrometer caliper and by taking photographs every other day. The mice were euthanized at the end of the studies (day 40 based on pilot study).

### Proteome profiler antibody array analysis

The mouse cytokine array panel an array kit (ARY006, R&D Systems) were used to analyze tissue lysates from pooled wide type tongue mucosa samples, *Tgfbr1/Pten* 2cKO tongue mucosa and 5 *Tgfbr1/Pten* 2cKO tongue SCC (*n* = 5 respectively) Average optical intensity of duplicate spots for each cytokine was normalized to the average of positive controls on the same chip using the Image J software (NIH, Bethesda, MD, USA).

### Flow cytometry analysis

To obtain single cell suspensions, tumor tissues of *Tgfbr1/Pten* 2cKO mice with or without PD-1 blockade antibody RMP1–14 treatment were processed using a gentle Macs dissociator and a murine tumor dissociation kit (Miltenyi Biotec). Single cell suspensions from spleens, lymph node, and blood were prepared according to a standardized protocol [51]. Wild type controls with the same dose of tamoxifen were used for flow cytometry analysis.

### Cell culture and RNAi

HNSCC cell lines CAL27 and FaDu were purchased from the American Type Culture Collection (ATCC, Manassas, VA) and genotype confirmed using STR methods. For the *in vitro* knockdown experiment, 2 TGFBR1 siRNA (siRNA5 and SiRNA6), 2 PTEN siRNA (siRNA5 and siRNA6) were used with negative control (Qiagen), and positive Cell Death siRNA (Qiagen). Validation of knock down efficiency using qPCR and Western blotting were performed as previously described [49, 50].

### Western blotting and quantitative real-time RT-PCR analysis

Western blotting and quantitative real-time RT-PCR analysis were performed as previously described [49].

### Human HNSCC tissue array

School and Hospital of Stomatology of Wuhan University Medical Ethics Committee approved this study, and informed consent was obtained from the patients before they underwent surgery. Custom made tissue microarray including 86 HNSCC, 12 oral epithelial dysplasia and 32 normal oral mucosa tissue samples were used for immunohistochemistry staining as previous described [4].

### Immunohistochemistry and immunofluorescence

Tumors from *Tgfbr1/Pten* 2cKO mice were dissected and fixed as previously described [31], and slides were stained with the appropriate antibody using a standard immunohistochemical and double immunofluorescence staining protocol as previously described [52].

### Scoring system, hierarchical clustering and data visualization

Whole slices were scanned and quantified of histoscore using an Aperio ScanScope CS scanner with a background substrate for each slice, quantification as previously described [53]. The hierarchical analysis was achieved using the Cluster 3.0 with average linkage based on Pearson's correlation coefficient [54].

### Statistical analysis

Statistical evaluations were undertaken with Graph Pad Prism version 5.0. One-way ANOVA followed by the post-Tukey multiple comparison tests were used to analyze the differences in protein levels, RNA levels and positive cells among each group. Student *t* test was used to analyze immunostaining of the difference between the PD-1 blockade group and vehicle group. Two-tailed Pearson's statistics was used for the correlated histoscore. The mean values ± SEM with a difference of *P* < 0.05 were considered statistically significant.

## SUPPLEMENTARY MATERIALS AND METHODS


