# Supplementary material for: PD-1 blockade attenuates immunosuppressive myeloid cells due to inhibition of CD47/SIRPα axis in HPV negative head and neck squamous cell carcinoma
Source: Oncotarget. 2015 Nov 7;6(39):42067–80. doi: 10.18632/oncotarget.5955 (PMC4747210; doi:10.18632/oncotarget.5955)
Supplement: Supplementary file 1 [file oncotarget-06-42067-s001.pdf]

SUPPLEMENTARY MATERIALS AND METHODS

Genetically modified mice

All experiments were conducted in accordance with the guidelines of the Institutional Animal Care and Use Committee of the Wuhan University. Time inducible tissue-specific *Tgfbr1/Pten* 2cKO mouse (*K14-Cre*$^{\text{ERtam+/-}}$; *Tgfbr1*$^{\text{flox/flox}}$; *Pten*$^{\text{flox/flox}}$) were maintained and genotyped according to published protocols [1, 2]. The details of *Tgfbr1* cKO HNSCC mouse (*K14-Cre*$^{\text{ERtam+/-}}$; *Tgfbr1*$^{\text{flox/flox}}$), *Pten* cKO HNSCC mice (K14-Cre$^{\text{ERtam+/-}}$; *Pten* $^{\text{flox/flox}}$) were previously described [1, 2]. All the mice were bred in the FVBN/CD1/129/C57 mixed background.

PD-1 antibody treatment

The *in vivo* MAb *anti* mPD1 antibody (RMP1–14) was purchased from BioXcell (West Lebanon, NH, USA) and was stored at 4°C at a concentration of 6.13 mg/ml. The working solution was diluted in PBS with a final concentration of 1 mg/ml immediately before use. The vehicle was used as a negative control for tumorigenesis experiments. After oral gavage of tamoxifen for 5 consequent days, the *Tgfbr1/Pten* 2cKO mice were randomly divides into control group (PBS, i.p. daily, n = 6 mice), 10 mg/kg RMP1–14 treated group (i.p. daily: n = 6 mice). RMP1–14 and vehicle treatment were performed 10 days after tamoxifen induction and the mice were observed for 18 days. Syngeneic control mice (*K14-Cre*$^{\text{ERtam+/-}}$; *Tgfbr1*$^{\text{flox/flox}}$; *Pten*$^{\text{flox/flox}}$) treated with the same dose of tamoxifen were used as controls (n = 6). For all animals, general inspection and monitoring were performed every day. The tumor sizes were measured using a micrometer caliper and by taking photographs every other day. The endpoint was determined according to a systematic evaluation by the veterinarian. The mice were euthanized at the end of the studies, and the tumors were fixed in paraffin overnight or frozen at −80°C for subsequent immunohistochemical or Western blot analysis.

Proteome profiler antibody array analysis

The proteome profiler^{TM} mouse cytokine array panel an array kit (ARY006, R&D Systems) consists of 40 different cytokine and chemokine antibodies spotted in duplicate onto a membrane. Tissue lysis from 5 pooled *Tgfbr1*$^{\textit{flox/flox}}$*; Pten*$^{\textit{flox/flox}}$ *K14Cre*$^{\textit{ERtam-/-}}$ tongue mucosa samples, 5 *Tgfbr1/Pten* 2cKO tongue mucosa samples and 5 *Tgfbr1/Pten* 2cKO tongue squamous cell carcinoma samples were used according to the manufacturer's instructions. Briefly, after blocking with 10% bovine serum albumin in Tris-buffered saline, the membranes were incubated with 250 µg (50 µg from each individual sample) of tissue lysis buffer in the presence of the supplied antibody cocktail. Following washes and incubation with a Streptavidin-HRP buffer, positive signals were revealed using ECL reagent. Positive (six spots) and negative controls (2 spots) were located in the corner of each array kit. A chemiluminescence substrate (Millipore, Billerica, MA, USA) was used to detect protein expression and the data were captured by multiple exposures to Kodak BioMax Light film. The arrays were scanned into a computer. For quantitation, after background subtraction, the average optical intensity of duplicate spots for each cytokine was normalized to the average of positive controls on the same chip using the Image J software (NIH, Bethesda, MD, USA).

Flow cytometry analysis

To obtain single cell suspensions, tumour tissues of mice (*K14-Cre*$^{\text{ERtam+/-}}$; *Tgfbr1*$^{\textit{flox/flox}}$*/Pten*$^{\textit{flox/flox}}$) with or without PD-1 blockade antibody RMP1–14 treatment were processed using a gentle Macs dissociator and a murine tumour dissociation kit (Miltenyi Biotec). Single cell suspensions from spleens, lymph node, and blood were prepared according to a standardized protocol [3]. Wild type controls with the same dose of tamoxifen were used for flow cytometry analysis. These cells were labelled with FITC-conjugated anti-mouse CD4, CD8 and CD11b, PE-conjugated anti-mouse PD-1 and Gr-1 (all from Becton Dickinson, Mountain View, CA); Percp-Cy5.5-conjugated anti-mouse F4/80, FITC-conjugated anti-mouse CD11c, PE-conjugated anti-mouse MHC-II, CD40, CD80 and CD86 (all from eBioscience, San Diego, CA); and isotype-matched IgG controls (eBioscience, San Diego, CA). The cells were analyzed on a FACScalibur flow cytometer equipped with CellQuest software, and gated by the side scatter and forward scatter filters. (Becton Dickinson, Mountain View, CA). Live cells were gated by 7AAD (Invitrogen) and populations were phenotyped as described above.

Cell culture and RNAi

HNSCC cell lines CAL27 and FaDu were purchased from the American Type Culture Collection (ATCC, Manassas, VA) and genotype confirmed using STR sequence. Cell lines were maintained in Dulbecco's modified Eagle's medium (DMEM)/F12, 10% fetal bovine serum (FBS), at 5% CO2 and 37°C in a humidified incubator. For the TGFBR1 and PTEN knockdown experiment, 2 TGFBR1 siRNA (siRNA5 and SiRNA6),

2 PTEN siRNA (siRNA5 and siRNA6) were transfected into appropriate cells using HiPerfect transfection reagent (Qiagen, Germantown, MD) with a final concentration of 5nM as previously described. All-star negative controls (Qiagen), confirmed to have no interference with other miRNAs, were used as negative controls. MAPK1 siRNA and Cell Death siRNA (Qiagen) were used as positive controls. For inhibition efficiency and target mRNA transcription studies, RNA was extracted 24 h after transfection and validated by qPCR. For protein extraction, cells were lysed 48 h after transfection. A combined knock down of TGFBR1 (siRNA5) and PTEN siRNA (siRNA6) were performed after validation of knock down efficiency as previous reported [1, 2].

Quantitative real-time PCR analysis

Quantitative real-time RT-PCR analysis was performed to evaluate the expression of representative cytokine and chemokine in *Tgfbr1*$^{flox/flox}$; *Pten*$^{flox/flox}$ *K14Cre*$^{ERtam-/-}$ tongue mucosa, *Tgfbr1/Pten* 2cKO tongue mucosa and *Tgfbr1/Pten* 2cKO tongue squamous cell carcinoma. RNA was extracted and reverse-transcribed into cDNA with random hexamers and Maloney murine leukemia virus reverse transcriptase (Takara, Kyoto, Japan). Real-time analysis was performed using Cd54, Csf1, Cxcl1, Cxcl2 and Ccl2-specific primers and probes with the ABI 7500 Sequence Detection System instrument and software (Applied Biosystems, Foster City, CA). Experiments were performed in triplicate for each sample.

Western blotting

Tumours that developed in *Tgfbr1/Pten* 2cKO mice were carefully dissected (n = 6, respectively). Cultured cells and tissue were lysed in a T-PER buffer containing1% phosphatase inhibitors and complete mini cocktail (Roche). The detailed immunoblotting procedures were described previously [4]. A total amount of 30 µg protein from each sample was denatured and then subjected to 12% SDS-polyacrylamide gel electrophoresis followed by transfer onto polyvinylidene fluoride membranes (Millipore Corporation, Bil-lerica, MA). Nest, the blots were using an enhanced chemiluminescence detection kit (West Pico, Thermo). GAPDH was detected on the same membrane and used as a loading control.

Human HNSCC tissue array

The School and Hospital of Stomatology of Wuhan University Medical Ethics Committee approved this study, and informed consent was obtained from the patients before they underwent surgery. Custom made tissue microarray including 86 HNSCC, 12 oral epithelial dysplasia and 32 normal oral mucosa tissue samples were used for immunohistochemistry staining as previous described [1].

Immunohistochemistry and immunofluorescence

Tumors from *Tgfbr1/Pten* 2cKO mice were dissected and fixed as previously described [4], and slides were stained with the appropriate antibody using a standard immunohistochemical staining protocol as previously described [5]. The bound antibodies were visualized using an appropriate biotin-conjugated secondary antibody and the Vectastain ABC Elite kit (Vector, Burlingame, CA, USA), using 3, 30-diaminobenzidine as the substrate (Sigma). For immunofluorescence, slides were hydrated in alcohol, washed three times in PBS, retrieved using sodium citrate in a pressure cooker, blocked with 2.5% bovine serum album in PBS buffer for 1 hour at 37°C and were then incubated with primary antibody overnight at 4°C. The next day, slides were incubated with fluorochrome conjugated secondary antibodies (Alexa 594 anti-rabbit and Alexa 488 anti-mouse; Invitrogen) and mounted in Vectashield with 4', 6-diamidino-2-phenylindole (DAPI; Vector Laboratories). Fluorescence images were then captured using a CLSM-310, Zeiss fluorescence microscope.

Scoring system, hierarchical clustering and data visualization

Whole slices were scanned using an Aperio ScanScope CS scanner (Vista, CA, USA) with a background substrate for each slice, and quantified using Aperio Quantification software (Version 9.1) for membrane, nuclear, or pixel quantification [6]. An area of interest was selected either in the epithelial or the cancerous area for scanning and quantification. The histoscore of membrane and nuclear staining was calculated as a percentage of different positive cells using the formula $(3+) \times 3 + (2+) \times 2 + (1+) \times 1$. The histoscore of pixel quantification was calculated as the total intensity/total cell number [1]. The threshold for scanning of different positive cells was set according to the standard controls provided by Aperio [1, 7].The expression scores were converted into scaled values centered on zero in Microsoft excel. Next, the hierarchical analysis was achieved using the Cluster 3.0 with average linkage based on Pearson's correlation coefficient [8]. Java TreeView 1.0.5 was used to visualize the results [9]. Finally, we arranged the clustered data and tissue samples on the horizontal axis and vertical axis respectively. Biomarkers with a close relationship are located next to each other.

Statistical analysis

Data analyses were performed using Graph Pad Prism version 5.0 for Windows (Graph Pad Software Inc, La Jolla, CA). One-way ANOVA followed by the post-Tukey multiple comparison tests were used to analyze the differences in protein levels, RNA levels and positive cells among each group. A unpaired *t* test was used to analyze immunohistochemical staining of the difference between the PD-1 blockade group and vehicle group. Two-tailed Pearson's statistics was used for the correlated expression of PD-1, PD-L1, CD11b, CD33, CD68 and CD163 after the confirmation of the sample with Gaussian distribution. The mean values ± SEM with a difference of $P < 0.05$ were considered statistically significant.

REFERENCES

1. Sun ZJ, Zhang L, Hall B, Bian Y, Gutkind JS, Kulkarni AB. Chemopreventive and chemotherapeutic actions of mTOR inhibitor in genetically defined head and neck squamous cell carcinoma mouse model. Clinical cancer research : an official journal of the American Association for Cancer Research. 2012; 18:5304–5313.
2. Zhang L, Sun ZJ, Bian Y, Kulkarni AB. MicroRNA-135b acts as a tumor promoter by targeting the hypoxia-inducible factor pathway in genetically defined mouse model of head and neck squamous cell carcinoma. Cancer letters. 2013; 331:230–238.
3. Trellakis S, Bruderek K, Hutte J, Elian M, Hoffmann TK, Lang S, Brandau S. Granulocytic myeloid-derived suppressor cells are cryosensitive and their frequency does not correlate with serum concentrations of colony-stimulating factors in head and neck cancer. Innate immunity. 2013; 19:328–336.
4. Bian Y, Terse A, Du J, Hall B, Molinolo A, Zhang P, Chen W, Flanders KC, Gutkind JS, Wakefield LM, Kulkarni AB. Progressive tumor formation in mice with conditional deletion of TGF-beta signaling in head and neck epithelia is associated with activation of the PI3K/Akt pathway. Cancer Res. 2009; 69:5918–5926.
5. Yu GT, Bu LL, Zhao YY, Liu B, Zhang WF, Zhao YF, Zhang L, Sun ZJ. Inhibition of mTOR reduce Stat3 and PAI related angiogenesis in salivary gland adenoid cystic carcinoma. American journal of cancer research. 2014; 4:764–775.
6. Huang CF, Zhang L, Ma SR, Zhao ZL, Wang WM, He KF, Zhao YF, Zhang WF, Liu B, Sun ZJ. Clinical significance of Keap1 and Nrf2 in oral squamous cell carcinoma. PloS one. 2013; 8:e83479.
7. Bian Y, Hall B, Sun ZJ, Molinolo A, Chen W, Gutkind JS, Waes CV, Kulkarni AB. Loss of TGF-beta signaling and PTEN promotes head and neck squamous cell carcinoma through cellular senescence evasion and cancer-related inflammation. Oncogene. 2012; 31:3322–3332.
8. Eisen MB, Spellman PT, Brown PO, Botstein D. Cluster analysis and display of genome-wide expression patterns. Proc Natl Acad Sci U S A. 1998; 95:14863–14868.
9. Saldanha AJ. Java Treeview—extensible visualization of microarray data. Bioinformatics (Oxford, England). 2004; 20:3246–3248.

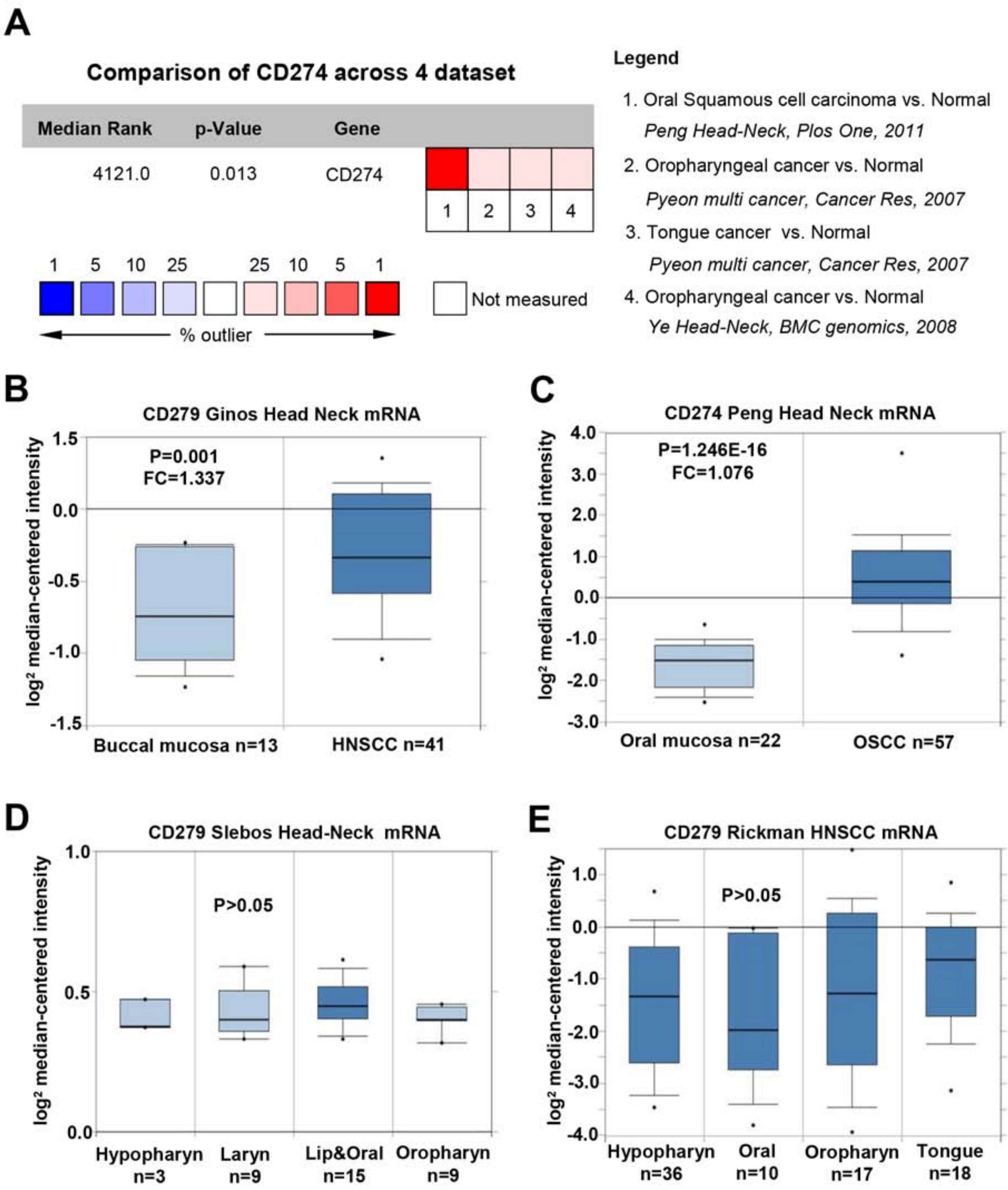

Supplementary Figure S1: *CD274, CD279* over expression in human HNSCC. A. meta-analysis of gene expression profiling for *CD274* (encoding *PD-L1*) in human HNSCC, where the colored squares indicated the median rank for *CD274* across each analysis. Significant increase of *CD279* (encoding PD-1) from Ginos's dataset **B.** and *CD274* mRNA level expression from Peng's dataset (as log2 median–centered ratio) **C.** in human HNSCC versus normal counterpart. The CD279 mRNA levels have no difference in various anatomic positions as shown in Slebos' datasheet **D.** as well as Rickman's datasheet **E.** Data retrieved from Oncomine database and present as mean ± SEM. Statistics by Oncomine.

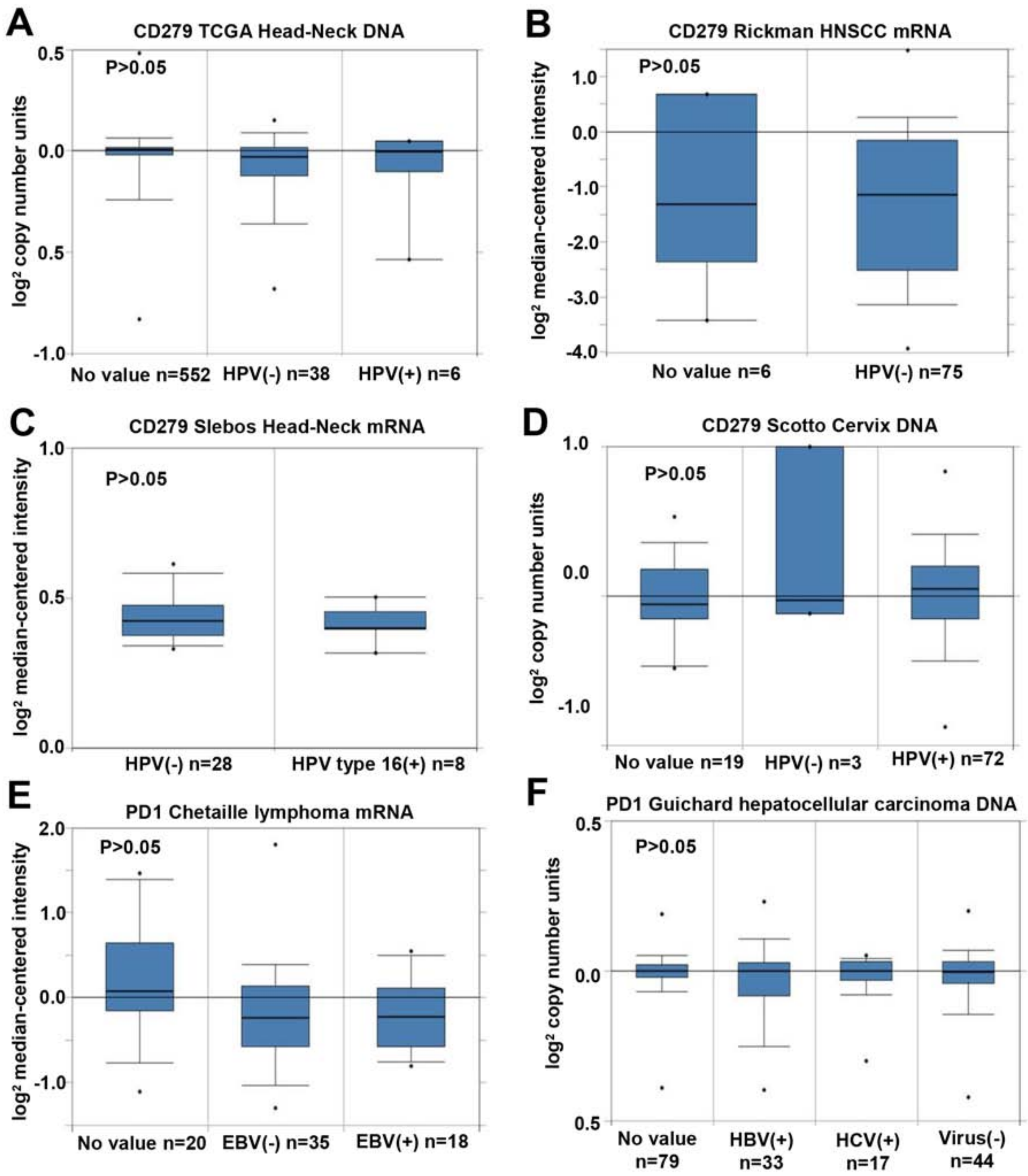

Supplementary Figure S2: *CD279* mRNA expression level is not related to viral infection in human cancer. A. data retrieved from TCGA head neck reveals no significant *CD279* DNA copy number difference between HPV+ and HPV- head neck cancer. Representative data shows no significant difference of *CD279* mRNA level between HPV+ and HPV- from Rickman's head neck cancer dataset **B.** or from Slebos' dataset (as log2 median –centered ratio) **C** and **D.** no significant difference of *CD279* DNA copy number level between HPV+ and HPV- cervical squamous cell carcinoma from Scotto's dataset. **E.** *CD279* mRNA level is no difference between EBV related lymphoma from Chetaille's dataset or HBV/HCV related hepatocellular carcinoma from Guichard's dataset (CD279 copy number unit). Data retrieved from Oncomine database and present as mean ± SEM. Statistics by Oncomine.

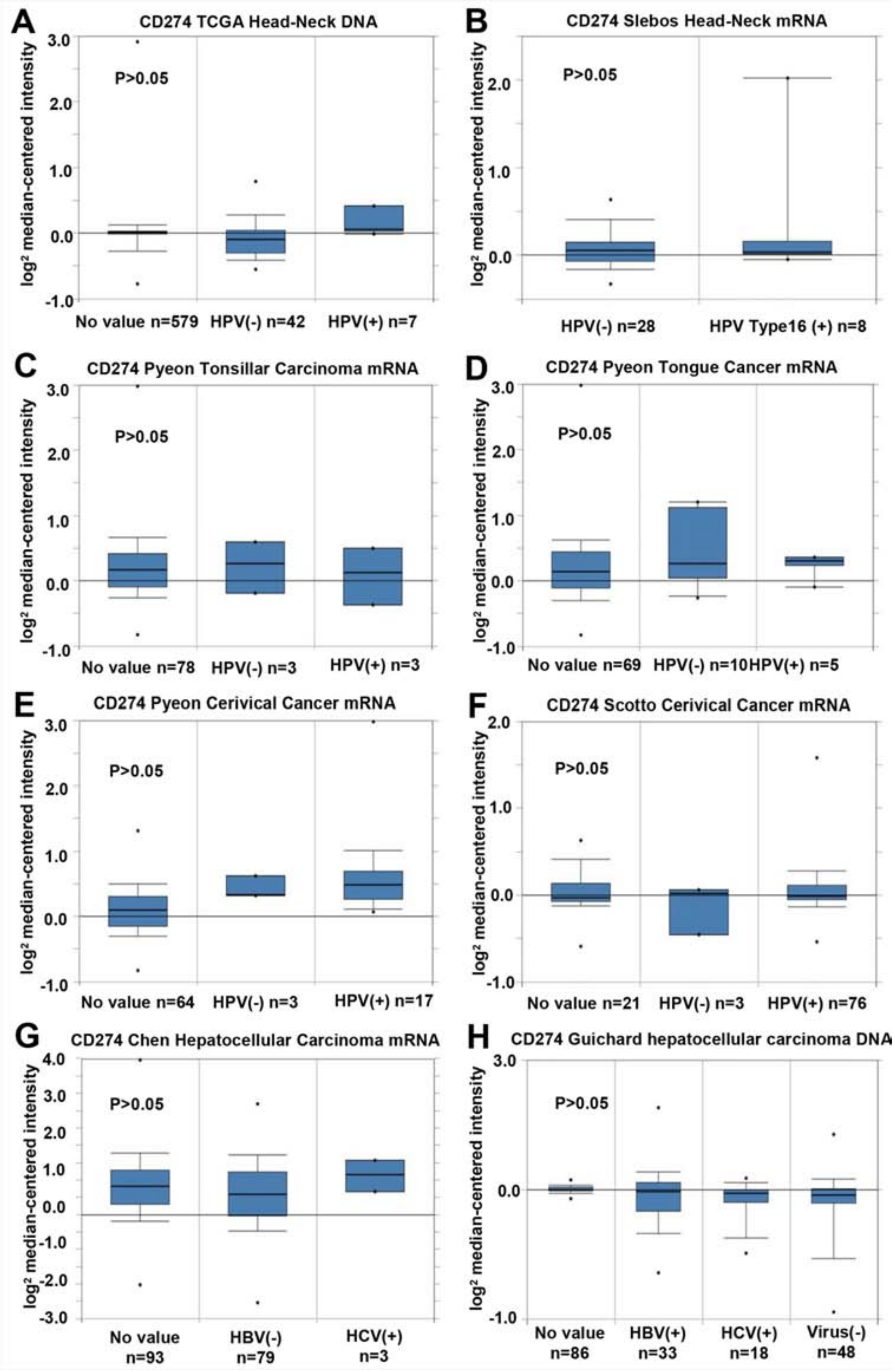

Supplementary Figure S3: *CD274* mRNA expression level and DNA copy number is not related to viral infection in human cancer. A. data retrieved from TCGA head neck reveals no significant *CD274* DNA copy number difference between HPV+ and HPV- head neck cancer. **B.** representative data shows no significant difference of *CD274* mRNA level between HPV+ and HPV- from Slebos's head neck cancer dataset. No significant difference of *CD274* mRNA level between HPV+ and HPV- tonsillar carcinoma **C.** tongue squamous cell carcinoma **D.** and cervical cancer **E.** from Pyeon's dataset. **F.** similar result from Scotto's cervical cancer dataset reveals *CD274* mRNA level is no difference between HPV+ and HPV- cervical cancer. CD274 mRNA level and DNA copy number unit have no difference from Chen's dataset **G.** as well as Chetaille's dataset **H.** on HBV/HCV related hepatocellular carcinoma. Data retrieved from Oncomine database and present as mean ± SEM. Statistics by Oncomine.

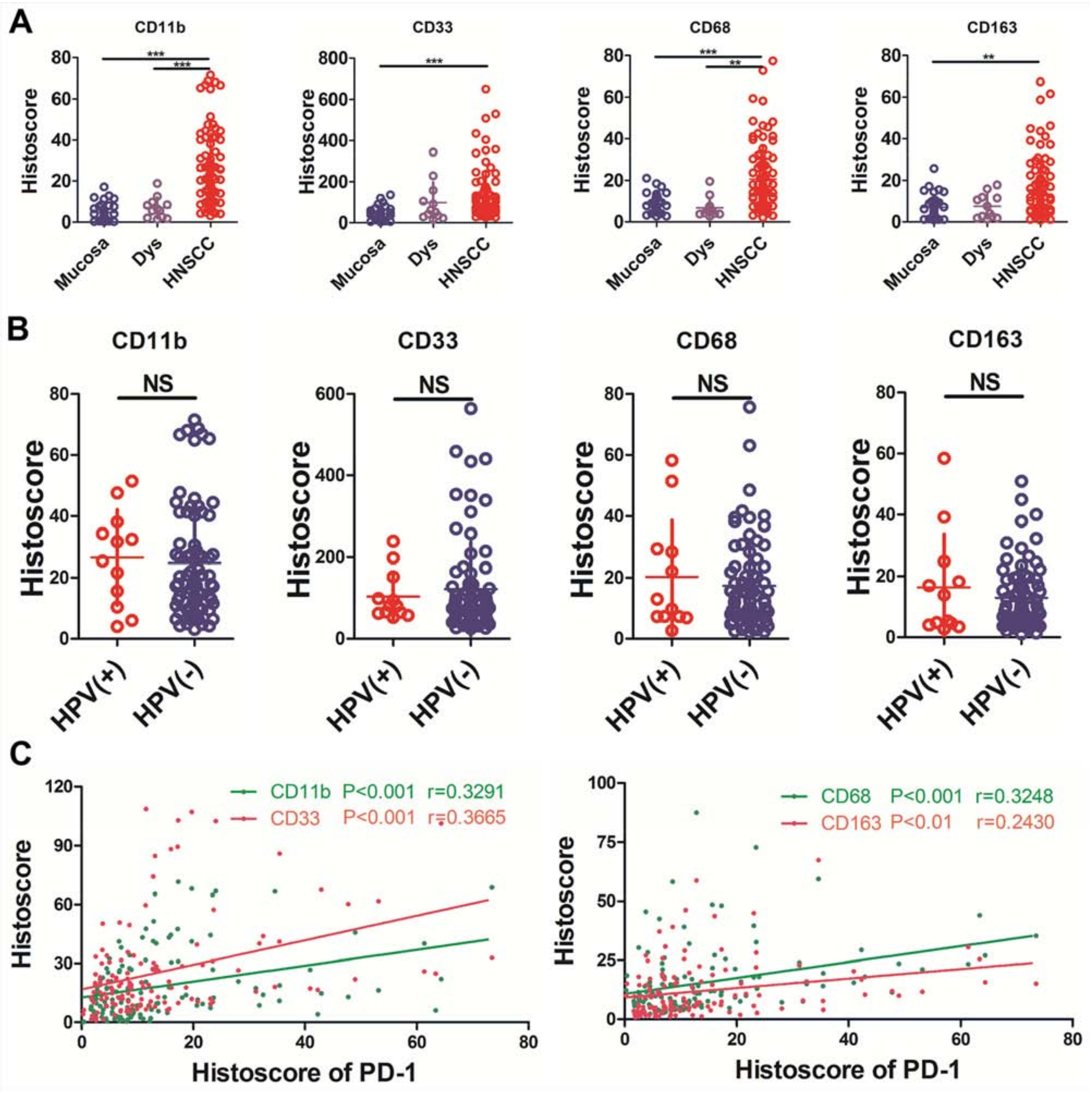

Supplementary Figure S4: Human HNSCC tissue array analysis reveals that increase PD-1 expression correlates with $CD11b^+CD33^+$ MDSCs and $CD68^+CD163^+$ TAMs. A. histoscore of immunohistochemistry staining of CD11b, CD33, CD68 and CD163 in human oral mucosa(Mucosa, $n = 32$), dysplasia (Dys, $n = 12$) as well as in head neck squamous cell carcinoma (HNSCC, $n = 74$) tissue (each dot presented as an independent sample, One way ANOVA with post Tukey test. **, $P < 0.01$; ***, $P < 0.001$). **B.** histoscore of CD11b, CD33, CD68 and CD163 in HPV+ ($n = 12$) and HPV- ($n = 74$) HNSCC. NS, No Significance. **C.** correlation and linear regression of PD-1 expression with CD11b/CD33 (left) and CD68/CD163 (right) in human oral mucosa, dysplasia and HNSCC. Each dot presented as an independent sample, Two-tailed Pearson's statistic including mucosa, dysplasia and HNSCC ($n = 130$).

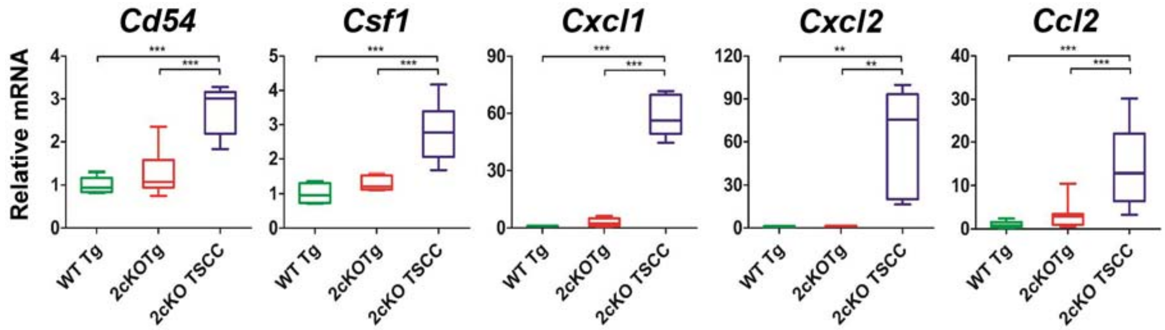

Supplementary Figure S5: Relative mRNA level of CD54, Csf1, Cxcl1, Cxcl2, Ccl2 of *Tgfbr1/Pten* 2cKO mice TSCC as compared with *Tgfbr1/Pten* 2cKO tongue and wide type tongue (*n* = 6 mice respectively, experiment repeated twice, One way ANOVA with post Tukey test. **, *P* < 0.01;*, *P* < 0.001).**

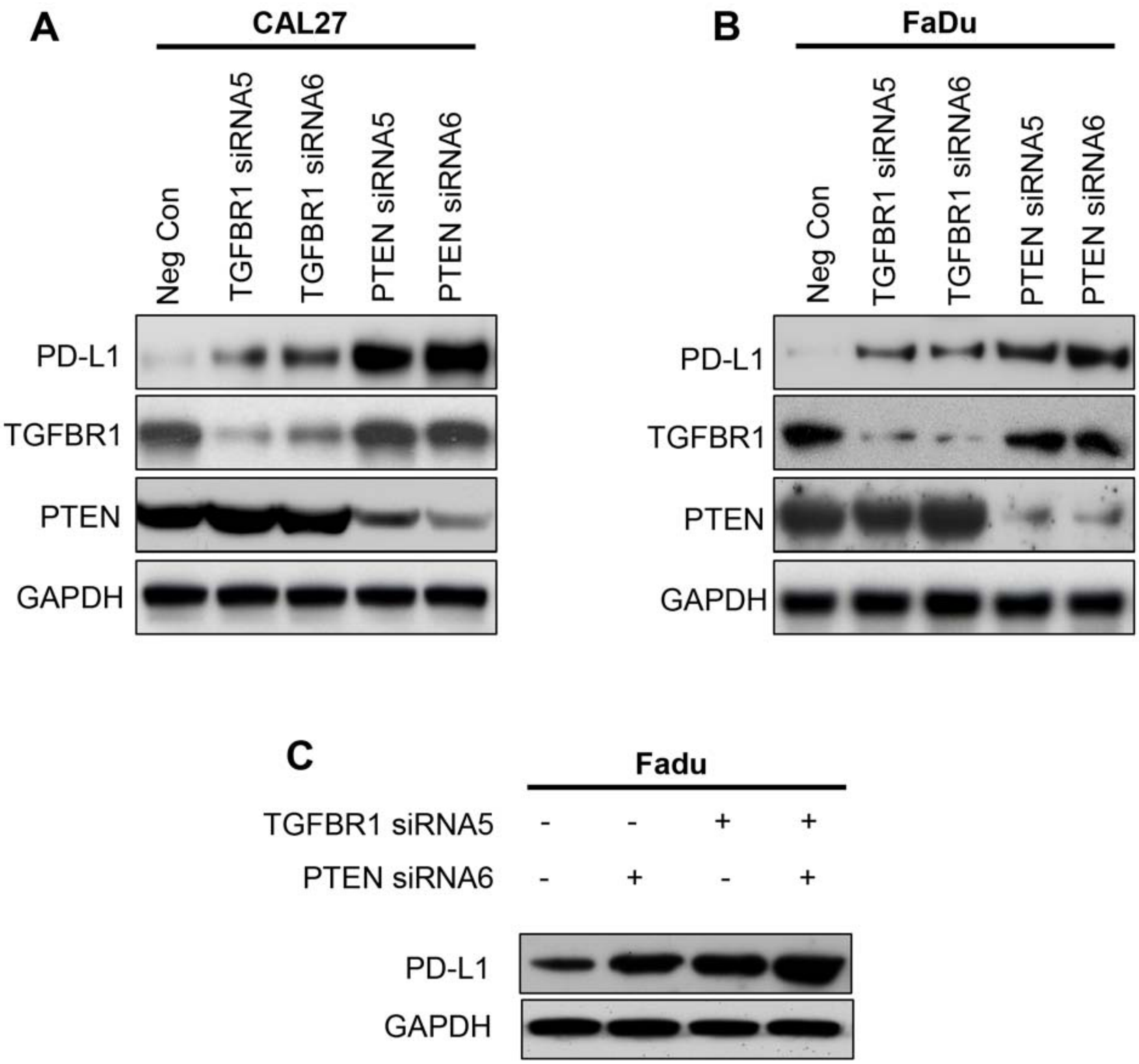

Supplementary Figure S6: *In vitro* knock down assay in CAL27 and FaDu cell lines. A. *In Vitro* knock down of TGFBR1 and PTEN using 2 siRNA sequence suggest effectively decrease of TGFBR1 and PTEN with increase of PD-L1 in CAL27 cell line as well as FaDu cell line **B.**. **C.** increase expression of PD-L1 by knock down TGFBR1, PTEN and combined TGFBR1 and PTEN in FaDu cell line using indicated siRNA (TGFBR1 siRNA5 and PTEN siRNA6).Neg Con, Negative control. Data show as representative photo of multiple exposures. Experiment repeated twice.

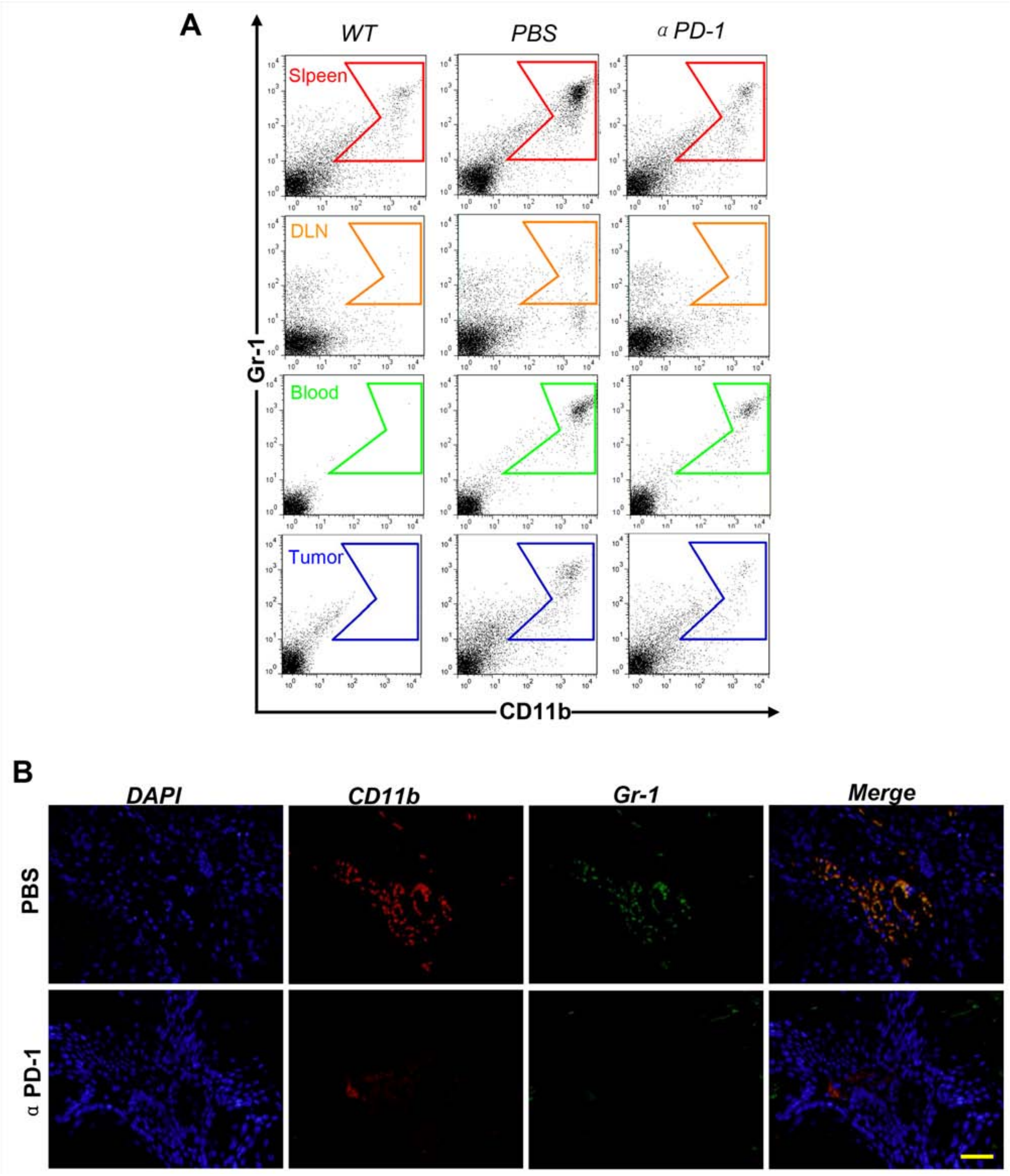

Supplementary Figure S7: αPD-1 significantly attenuate increased $CD11b^+Gr1^+$ MDSCs in HNSCC bearing *Tgfbr1/Pten* 2cKO mouse model. A. representative flow cytometry profiles shows increased CD11b+Gr1+ in spleen, draining lymph node (DLN), blood and tumor of HNSCC bearing *Tgfbr1/Pten* 2cKO mouse (PBS treatment, middle) as compared with wide type (WT) mice (n = 6 mice respectively) **B.** representative double immunofluorescence staining of CD11b+ Gr1+ cell population in mice HNSCC with or without αPD-1 treatment as well as wide type (WT) mice. Scale bar, 50um.

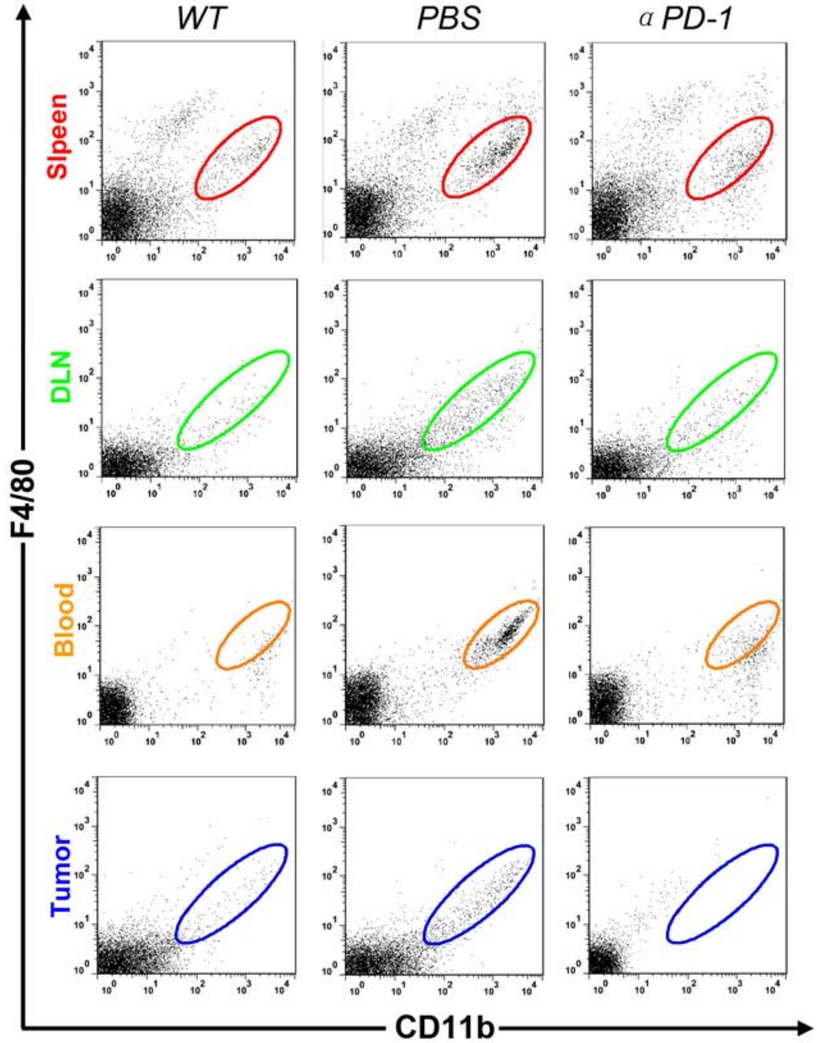

Supplementary Figure S8: αPD-1 significantly attenuate increased CD11b$^+$F4/80$^+$ TAMs in HNSCC bearing *Tgfbr1/Pten* 2cKO mouse model. A, Reprehensive flow cytometry profiles show single cell suspension from spleen, draining lymph node (DLN), blood, tumor or oral mucosa of HNSCC bearing mouse treated with αPD-1 or PBS and wide type (WT) mice were stained with anti-CD11b and anti-F4/80 antibody and percentage of positive cells analyzed by flow cytometry.

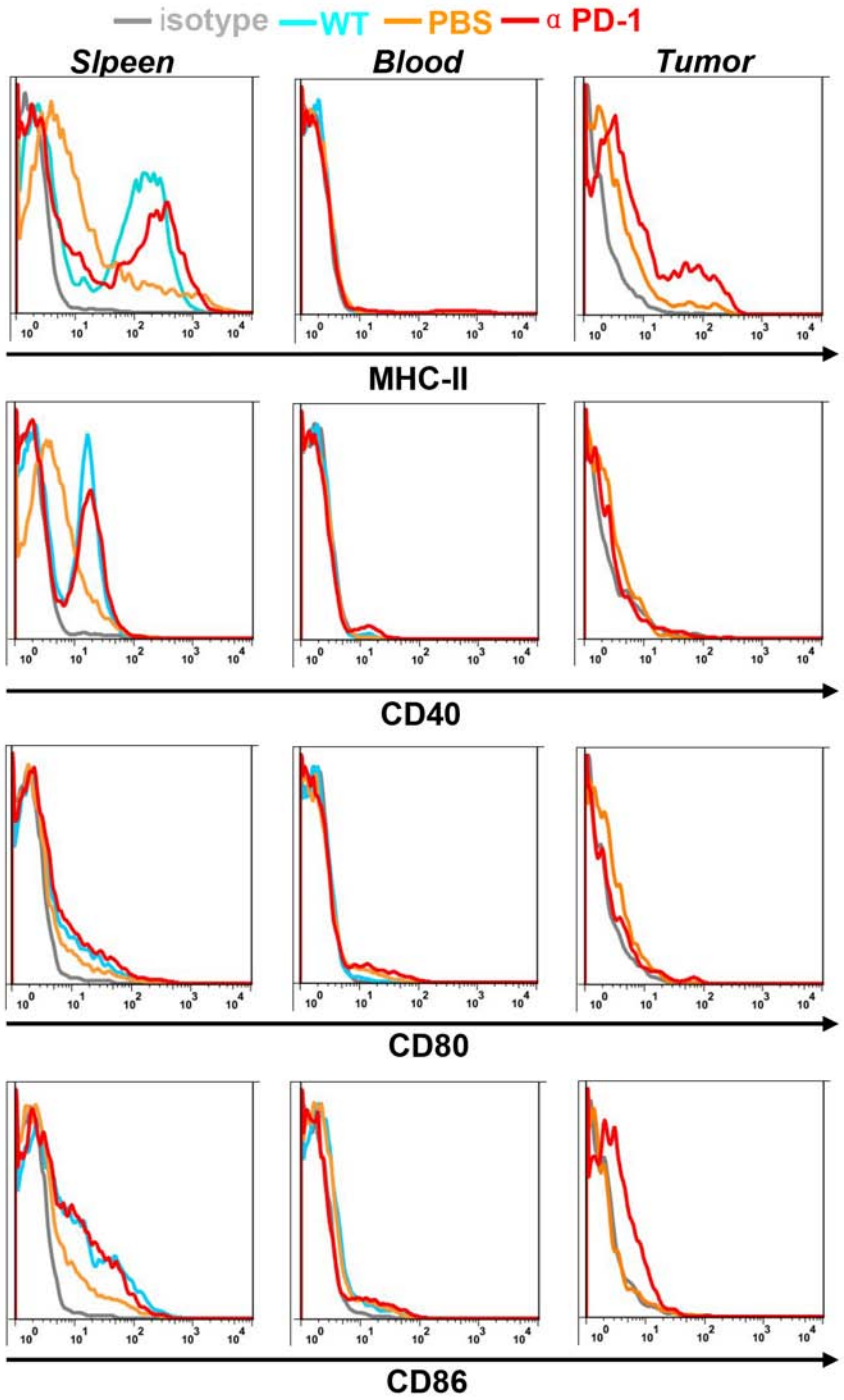

Supplementary Figure S9: Inhibition of PD-1 significantly increase mature DCs in HNSCC mouse model. Representative photo showed increase MHC-II$^+$, CD40$^+$, CD80+ or CD86$^+$ staining cell population in spleen, draining lymph nodes (DLN), blood and tumor of αPD-1 group as compared with PBS group and wide type (WT) group using flow cytometry.
